# Spatial distribution and associated factors’ of early marriage among reproductive age women in Ethiopia: a secondary data analysis of Ethiopian Demographic and Health Survey 2016

**DOI:** 10.1186/s12905-020-01103-5

**Published:** 2020-12-07

**Authors:** Zemenu Tadesse Tessema

**Affiliations:** Department of Epidemiology and Biostatics, Institute of Public Health College of Medicine and Health Science, University of Gondar, Gondar, Ethiopia

**Keywords:** Early marriage, Childhood marriage, Ethiopia

## Abstract

**Background:**

Besides, the presence of national law, the country has to set up its own mid-term and long term goals to bring about a significant reduction in child marriages in Ethiopia. As my search concerned, there is no study conducted on the spatial distribution of early marriage in Ethiopia. Determining the spatial distribution of early marriage and factors associated is important for government, other concerned bodies, program implementers, and policy developers to end up early childhood marriage. Thus, this study aimed to assess the spatial distribution and associated factors of Early marriage among reproductive-age women in Ethiopia.

**Methods:**

This study analyzed retrospectively a cross-sectional data on a weighted sample of 11,646 reproductive age women after requesting from Ethiopian Demographic and Health Survey 2016. ArcGIS and SaTScan software were for spatial analysis. Multiple logistic regression analysis was used to identify factors associated with early marriage. Finally, variables with a p-value of less than or equal 0.05 were considered as statistically significant.

**Results:**

In this analysis, about 62.8% (95% CI 61.9, 63.74%) of the study participants were married before they reached 18 years. The overall median age at first marriage was 17.1 with IQR 5 years. The high clustering of early marriage was located in Amhara, Afar, and Gambella Regions. In spatial Scan statistics, 87 clusters (RR = 1.28) significant primary clusters were identified. The associated factors of early marriage were lesser among women’s attending primary (AOR = 0.60; 95% CI 0.51, 0.71), secondary (AOR = 0.19; 95% CI 0.13, 0.26) and tertiary education (AOR = 0.11; 95% CI 0.07, 0.18). Similarly, women found in Addis Ababa were at a lesser risk of early marriage compared to other regions of the country.

**Conclusion:**

Marriage below age 18 is high in Ethiopia. High-risk area of early marriage was located in Amhara, Afar, and Gambella. Governmental and non-governmental organizations should design an effective intervention in these regions to reduce Early marriage. Therefore, providing educational opportunities to young girls was important in addition to inhibiting the marriage of girls under 18 years.

## Background

Early marriage is defined as the marriage of a girl < 18 years of age and is a common phenomenon worldwide [1]. According to the United Nations Children’s Fund (UNICEF), Each year, 12 million girls are married before the age of 18 years [2]. The problem is highly prevalent in Asia (45%) followed by sub-Saharan Africa (39%), Latin America (23%), and 18% in the Middle East and North Africa [3]. As an illustration, in Africa, the prevalence of early marriage was 31.4% in Zambia [4]. Similarly, in Ethiopia, the percentage of women marrying before age 18 has declined slightly since 2011 from 63 to 58%. During the same period, the median age at first marriage among women age 25–49 has increased from 16.5 years to 17.1 years [5].

Childbearing below the age of 18 years is associated with a higher rate of mortality, eclampsia, postpartum hemorrhage, HIV infection, malaria, and obstructed labor [6, 7]. Besides, early marriage is associated with lower levels of schooling for girls, higher intimate partner violence, and poor maternal and child nutrition status [6]. The probability of being stunted and wasting is higher among children born from early married women [8]. The consequence of early marriage is not limited to the mother and her child, it has also social, economic, and political implications [9].

Despite the presence of national laws in Ethiopia, its marriage of girls < 18 years of age is common and it affects a number of girls [10]. The problem may worsen when it exists with a high prevalence of HIV and other sexually transmitted diseases, malnutrition, cervical cancer, and others [11]. The governments of Ethiopia have adopted strategies to end the practice and investments are being made to that effect, including by promoting girls’ education and sexual and reproductive health and rights. But ending child marriage requires a multifaceted approach focused on the girls, their families, the community, and the government.

In Ethiopia, several studies identified that education, harmful traditional practice, income, family size, media exposure, and culture of the community were the significant factors associated with early marriage [10, 12–18]. So far different studies in Ethiopia have been done to identify the factors associated with early marriage. But, the spatial pattern of early marriage has not been done before. Identifying the spatial pattern of early marriage in Ethiopia can help health planners and policymakers to develop target interventions to decrease early marriage.

The research hypothesis of this study is that is there any relationship between the outcome variable (early marriage) and socio-economic and demographic variables in Ethiopia. The other hypothesis is that whether the early marriage was randomly distributed or not across the county.

Therefore, besides the presence of national law, the country has to set up its own mid-term and long term goals to bring about a significant reduction in child marriages in Ethiopia. To achieve this, showing the spatial pattern and its factors associated are important for government, other concerned bodies, program implementers, and policy developers to end early childhood marriage. Thus, the aim of this study was to assess the spatial patterns and associated factors of Early marriage among reproductive-age women in Ethiopia.

## Methods

### Study area and period

The study was conducted in Ethiopia, located in the horn of Africa. The area had nine regional states and two city administrations. The detail of the study area was found in our previously published articles [19]. The study period was January, 18/2016 to June, 27/2016.

### Data source

The study was based on the Ethiopian Demographic and Health Survey (EDHS) 2016 data set. Approval letter for the use of this data was gained from the Measure DHS program which is publicly available at www.measuredhs.com.

### Sampling procedure and sample size

The survey covered all the nine regions and the two city administrations of Ethiopia and participants were selected through a stratified two-stage cluster sampling technique. The full details of the methods and procedures used for the collection of the EDHS data have been published elsewhere [5]. The survey collected information was from a nationally representative sample of 16,683 women aged 15–49 years. Finally, 11,646 eligible women were included in this study five years preceding the survey which was nested within 643 clusters across the country.

### Source and study population

**Fig. 1:**
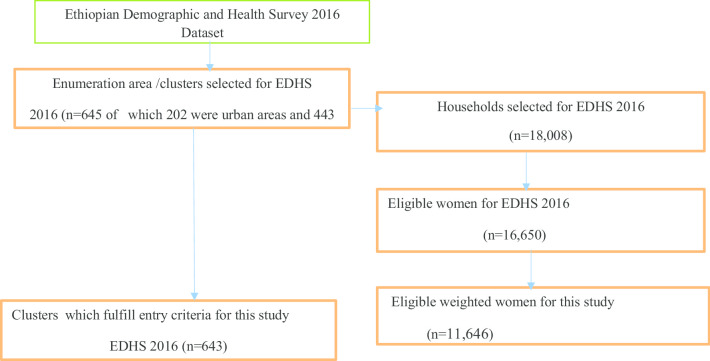
Sampling procedures of age at first marriage among Ethiopia women age 15–49 using EDHS 2016 data

The source population was all reproductive-age women in Ethiopia within five years preceding the survey. The study population was all reproductive-age women in the selected enumeration areas within five preceding the survey. A total of 18,008 households were selected and 16,650 were successfully interviewed. A total of 11,646 women who had married five years preceding the survey were included in this analysis (Fig. 1).

### Variables o f the study

The outcome variable for this study was the age at first marriage (binary) either below 18 or 18 and above. The variables that may influence early marriage include age, religion, respondents' highest education attainment, educational status of husbands/parents, occupational status of respondents, occupational status of parents, media exposure, and household wealth status, residence, and region [10, 12–18].

Media exposure was calculated by aggregating three variables such as watching television, listening to the radio, and reading newspapers. Then divided as having media exposure if a mother has been exposed to at least one of the three and not if she had no exposure to all of the source.

Wealth index was generated households were given scores based on the number and kinds of consumer goods they own, ranging from a television to a bicycle or car, in addition to housing characteristics such as the source of drinking water, toilet facilities, and flooring materials. These scores are derived using principal component analysis [5]. National wealth quintiles were compiled by assigning the household score to each usual (de jure) household member, ranking each person in the household population by her or his score, and then dividing the distribution into five equal categories, each comprising 20% of the population. Accordingly, the household wealth index was categorized as (1 = Poorest; 2 = Poorer; 3 = Middle; 4 = Richer; 5 = Richest). For this study, for ease of interpretation and analysis wealth index recorded as [Poorest and Poorer = poor (1), Middle = Middle (2), Richer, and Richest = rich (3)].

### Data collection procedure, tools, and quality control

The data was obtained from Individual Records (IR) file EDHS 2016. The EDHS 2016 was used as a structured and pre-tested questionnaire for data collection The 2016 EDHS data collectors used tablet computers to record responses during the interview. The tablet was equipped with Bluetooth technology to enable remote electronic transfer of files for this study the detail is found at [5].

### Spatial analysis

Spatial autocorrelation measures how much close clusters are in comparison with other close clusters. Positive spatial autocorrelation is when similar values cluster together on a map. Negative spatial autocorrelation is when dissimilar values cluster together on a map. Hot spot analysis identifies statistically significant hot spots and cold spots using the Getis-Ord Gi* statistic. Interpolation is a procedure used to predict the values of cells at locations that lack sampled points. Spatial scan statistics performed using SatScan software to identify the primary, secondary, terciart….etc. most significant clusters using Bernoulli-based model. The detail for each spatial analysis is found in our previously published article [20].

### Statistical analysis

The data was cleaned using appropriate data set using DHS guideline statistics. Both descriptive and analytical statistics were done. Bivariable and multiple logistic regression were done to see the association and between early marriage and covariates. In the bivariable analysis that had a p-value less or equal to 0.2 were taken for further analysis for the final model. In the final multiple logistic regression model result that had a p-value less or equal to 0.05 were declared for the significant association between early marriage and covariates. Both the crude odds ratio (COR) and adjusted odds ratio(AOR) with its 95% confidence interval were reported.

## Results

A total of 11,646 study participants were included in this study. Of these, 7322 of them or 62.8% (95% CI 61.9, 63.74%) of the study participants were married before they reached 18 years. The overall median age at first marriage was 17.1 years with IQR 5. The majority, 9544 (88.95%), of the respondents were in rural areas. More than half 7059 (60.61%) of respondents had no formal education. Near to three-fourth, 7782 (77.9%), of the respondents had exposure to mass media (Table 1).

**Table 1:** socio-demographic and economic characteristics of the study participants, EDHS 2016

Variables	Frequency n = 11,646	Percentages
Age specific marriage		
10	145	0.92
11	201	1.28
12	401	2.56
13	750	4.78
14	1204	7.68
15	2345	14.65
16	1984	12.65
17	1722	10.87
≥ 18	7322	62.87
Age at marriage		
Less than 18 year	4324	37.13
18 years and above	7322	62.87
Mother’s age		
< 20	1237	10.61
20–34	6268	53.81
35–49	4142	36.56
Religion		
Orthodox	4970	42.67
Muslim	3906	33.54
Protestant	2498	21.45
Others^a^	271	2.33
Residence		
Urban	2102	18.05
Rural	9544	81.95
Region		
Tigray	487	7.23
Afar	108	0.93
Amhara	2888	24.79
Oromia	4433	38.08
Somalia	358	3.07
Benishangul Gumuz	125	1.07
SNNP	12,310	19.08
Gambela	34	0.79
Harari	29	0.25
Addis Ababa	451	3.88
Dire Dawa	63	0.54
Mother’s educational status		
Unable to read and write	7059	60.61
Primary education	3351	28.77
Secondary education	764	6.56
Higher education	473	4.06
Husband educational status		
Unable to read and write	4763	46.59
Primary education	3772	39.90
Secondary education	975	9.54
Higher education	713	6.97
Mother’s occupation		
Not working	5968	48.76
Working	5679	51.24
Husband occupation		
Not working	807	5.16
Working	9416	94.84
Media exposure		
No media exposure	2583	22.10
Has media exposure	7782	77.90
Wealth index		
Poor	4504	38.67
Middle	2324	19.95
Rich	4819	41.38

^a^Others represent catholic and traditional religion follower

### Spatial distribution of early marriage in Ethiopia

#### Spatial autocorrelation

**Fig. 2:**
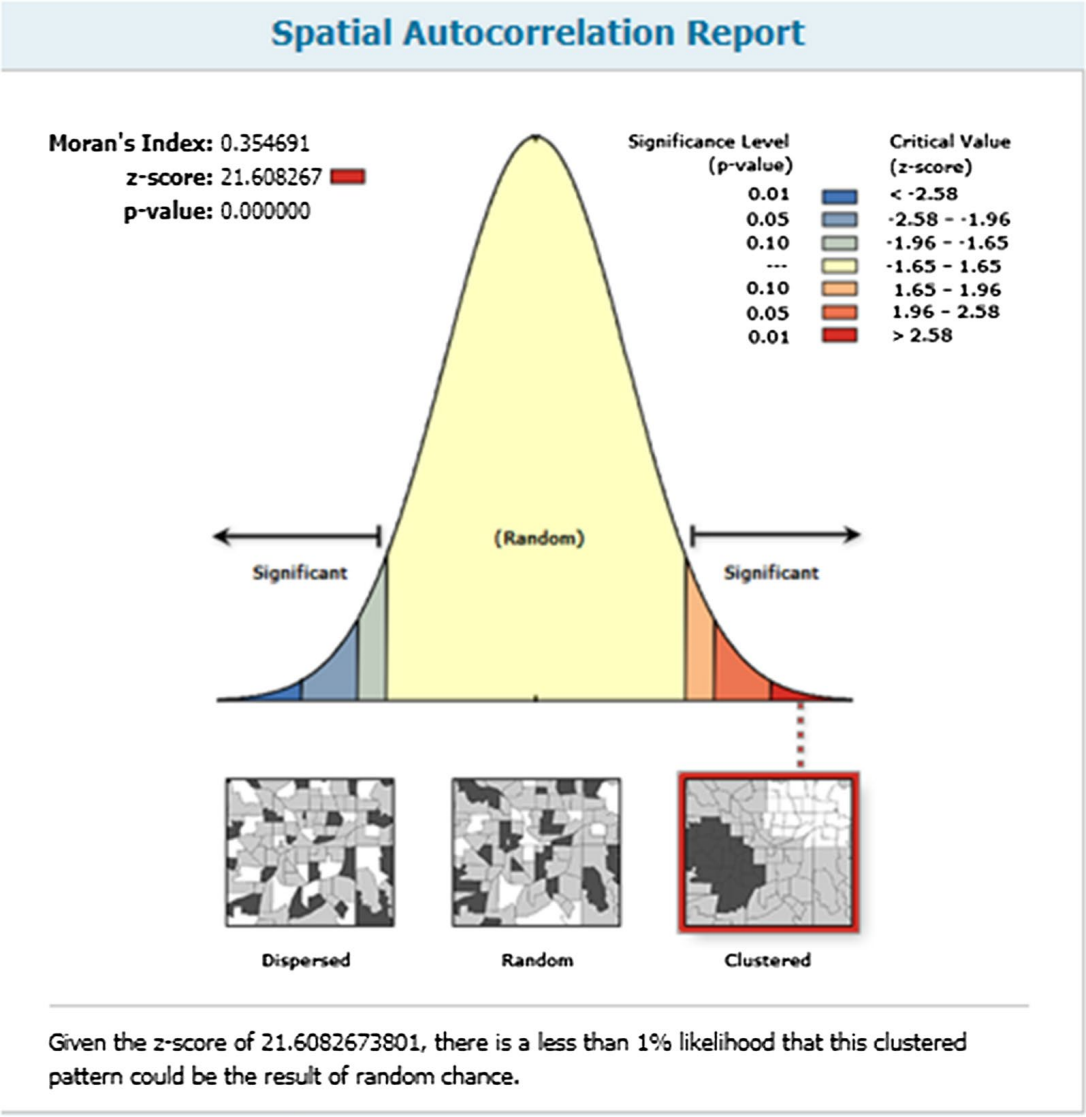
Spatial autocorrelation of Early marriage in Ethiopia among the reproductive age group in EDHS 2016

The spatial distribution of Early marriage in Ethiopia was non-random in the EDHS 2016 dataset. The global Moran’s I value was 0.354 (*p* value < 0.001) and Z-score 21.6 in the 2016 Ethiopian Demographic and Health Survey (Fig. 2).

### Incrementa spatial l autocorrelation early marriage among reproductive-age women in Ethiopia

**Fig. 3:**
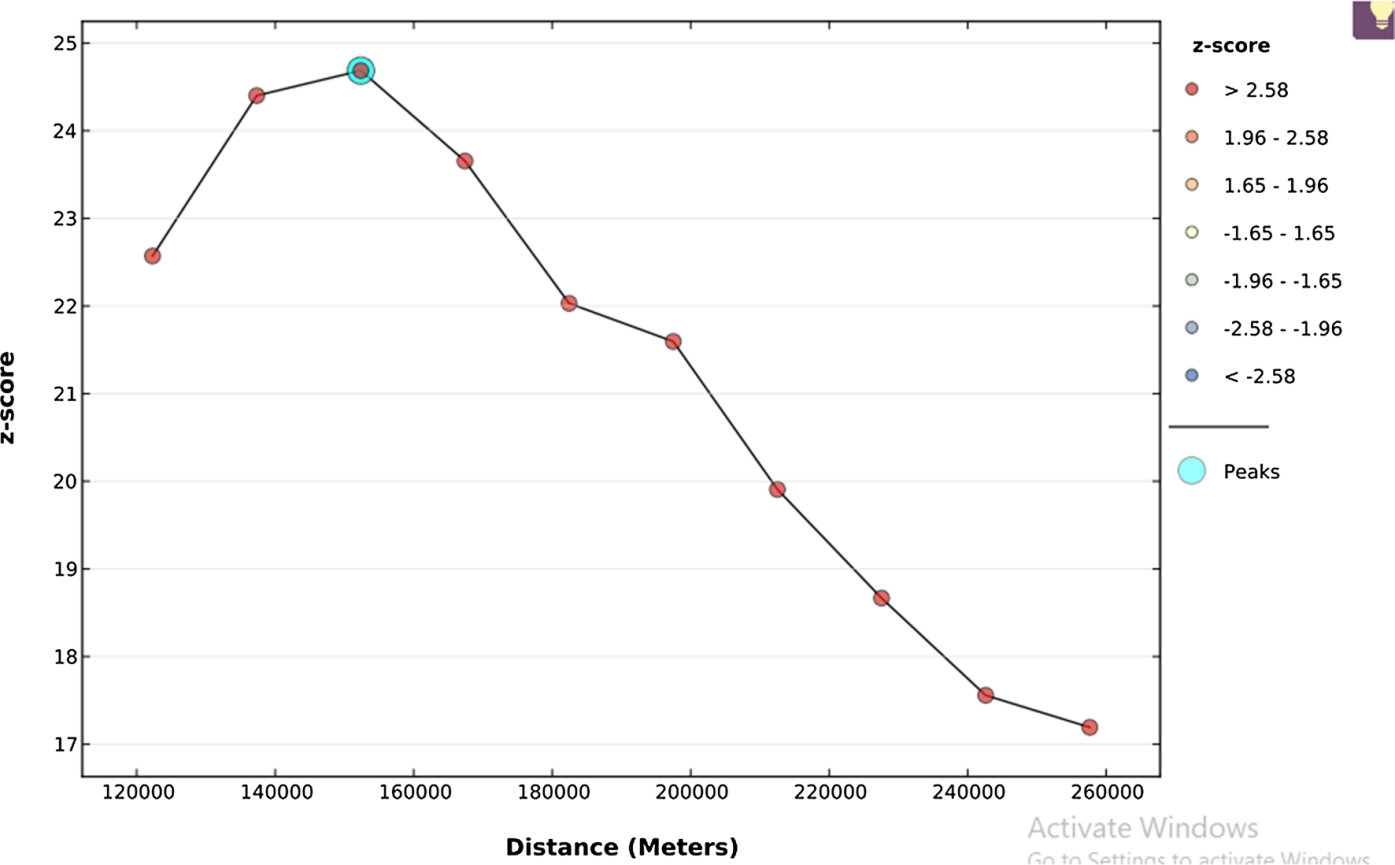
The spatial autocorrelation of early marriage among reproductive age group women in Ethiopia by a function of distance

To determine spatial clustering for early marriage, global spatial statistics were estimated using Moran’s I value. As shown in the figure below a statistically significant z-scores indicate at 151.3 km distances where spatial processes promoting clustering are most pronounced. The incremental spatial Autocorrelation indicates that a total of 10 distance bands were detected with a beginning distance of 121,813 m (Fig. 3).

### Hot spot (Getis-Ord Gi) analysis

**Fig. 4:**
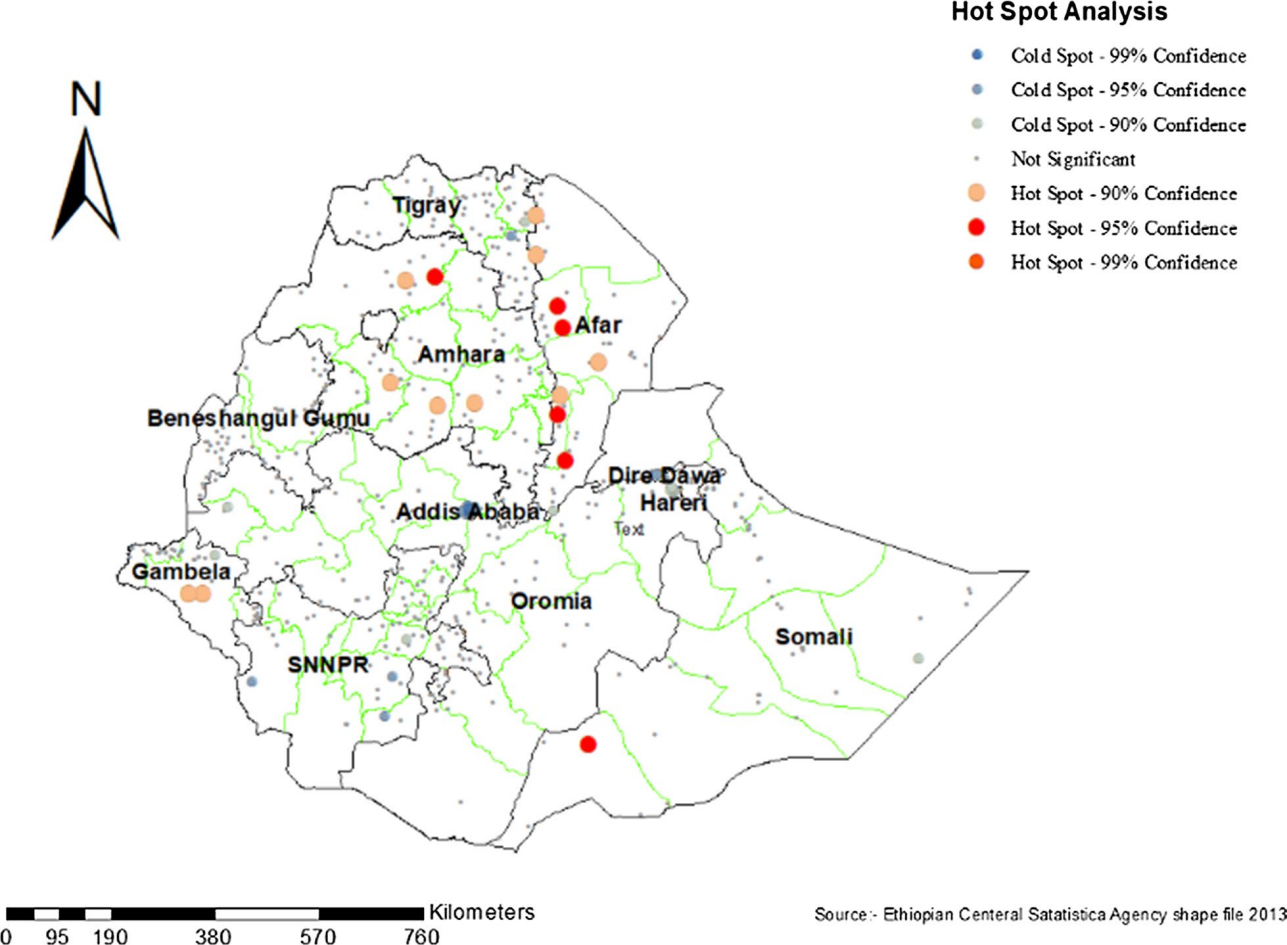
Hot spot analysis of Early marriage among women within 5 years preceding the survey in Ethiopia, 2016

As shown in the figure below, the red color indicates the more intense clustering of high (hot spot) proportion early marriage preceding the survey period. A high proportion of early marriage was located at the Amhara, Afar, and Gambella region of Ethiopia. Whereas, Amhara, SNNPR, and Addis Ababa regions of Ethiopia were less risk area. Amhara Region was included in both high proportion and fewer risk areas (Fig. 4).

### Spatial Sat Scan analysis of early marriage among women across regions of Ethiopia, 2016

**Fig. 5:**
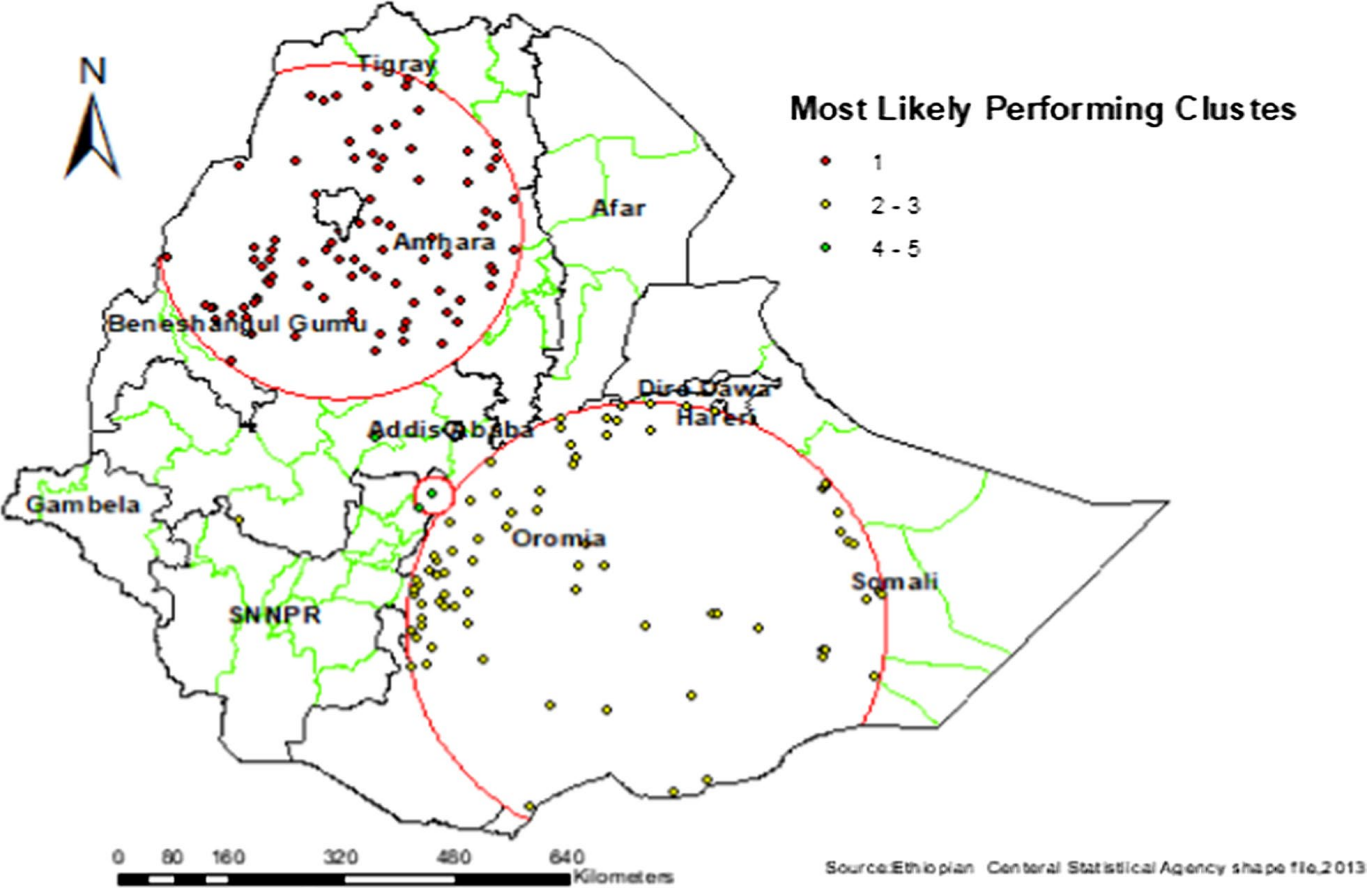
SatTscan analysis result of Early marriage among Reproductive age women in Ethiopia EDHS 2016

Most likely (primary clusters) and secondary clusters of early marriage were identified. A total of 163 (87 primary and 76 secondaries) significant clusters were identified. The primary clusters' spatial window was located in the Amhara Tigray and Benishangul regions, which was centered at 11.66 N, 37.31 E with a 254.88 km radius, and Log-Likelihood ratio (LLR) of 126.18, at *p* < 0.001. It showed that women within the spatial window had 1.28 times higher risk of early marriage than women outside the window. The secondary clusters' spatial window was typically located in the Somali and Oromia regions. Which was centered at 6.30 N, 41.25E with 340.06 km radius, and LLR of 18.95 at *p* value < 0.001 It showed that women within the spatial window had a 1.11 times higher risk of early marriage than women outside the window (Fig. 5, Table 2).

**Table 2:** SaT Scan analysis of Early marriage among women in the last five years in Ethiopia, 2016

Cluster type	Significant enumeration areas (clusters) detected	Coordinates/radis	Populations	Cases	RR	LLR	*P *value
Primary	169, 73, 431, 158, 516, 382, 167, 512, 292, 163, 361, 456, 403, 429, 132, 24, 259, 109, 602, 3, 541, 327, 640, 120, 515, 415, 548, 279, 386, 615, 498, 375, 152, 38, 312, 627, 638, 199, 474, 206, 533, 246, 545, 628, 322, 559, 176, 482, 531, 52, 494, 36, 229, 80, 150, 218,350, 66, 10, 183, 184, 296, 460, 591, 612, 401, 504, 137, 267, 425, 364, 244, 542, 35, 354, 478, 510, 258, 616, 617, 300, 188, 256, 320,136, 410, 340, 200, 392, 551	(11.699828 N, 37.313042 E) / 254.88 km	2756	2803	1.28	126.18	< 0.001
Secondary	480, 187, 318, 286, 289, 556, 472, 394, 452, 278, 377, 123, 422, 562, 520, 34, 213, 319, 358, 85, 164, 518, 208, 26, 529, 619, 405, 245, 468, 576, 313, 122, 524, 476, 365, 372, 589, 316, 12, 391, 438, 95, 412, 198, 578, 445, 600, 492, 522, 398, 308, 506, 171, 634, 497, 7, 71, 216, 232, 521, 215, 588, 553, 148, 32, 149, 138, 408, 458, 543, 333, 490, 21, 92, 49, 93, 453, 513	(6.300866 N, 41.252617 E) / 340.06 km	2691	1833	1.11	61.2918.95	< 0.001

### Interpolation of early marriage in Ethiopia

**Fig. 6:**
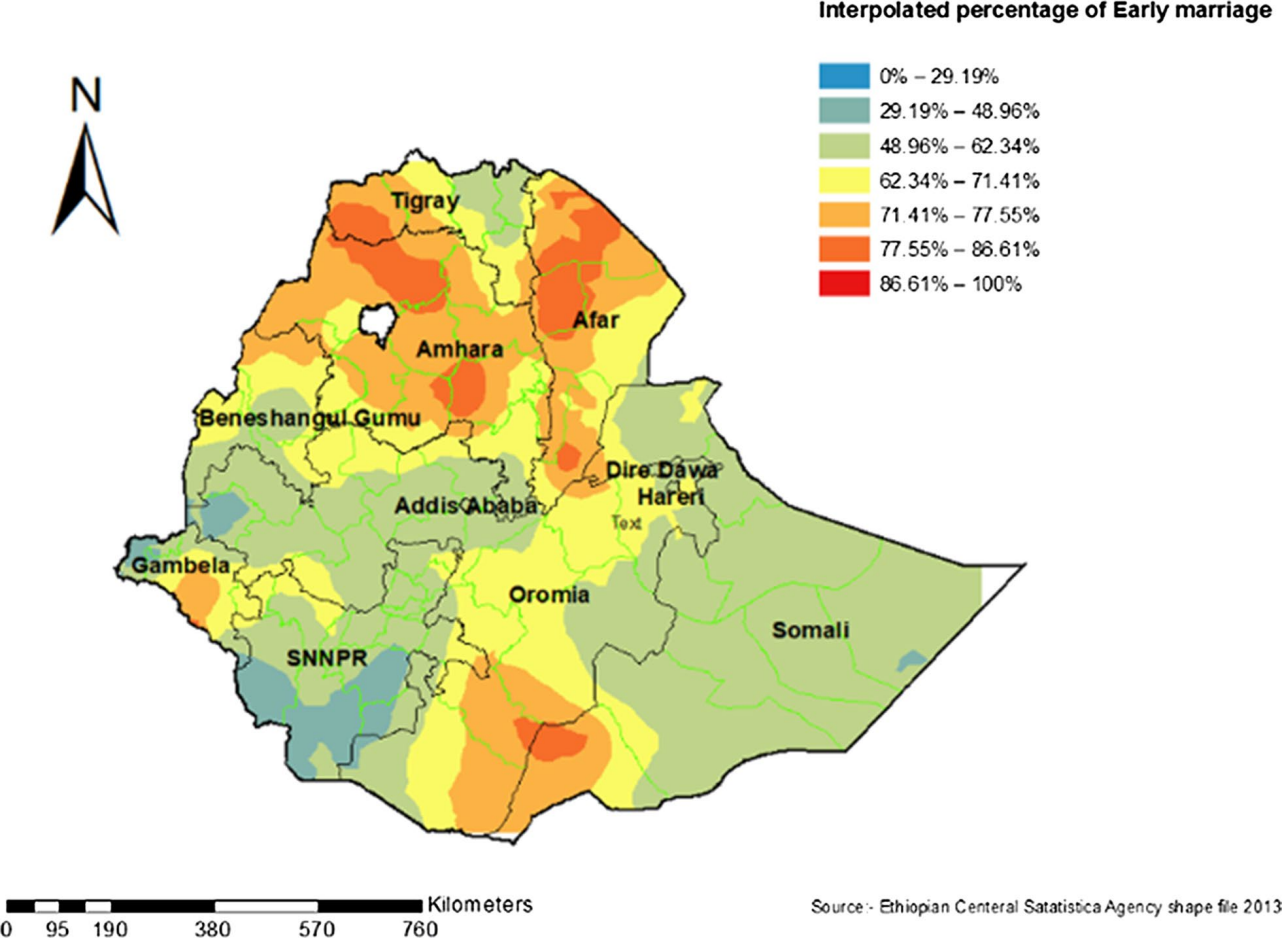
Interpolation of Early marriage among reproductive-age women in Ethiopia, 2016

The predicted early marriage over the area increases from green to red-colored areas. The red color indicates high-risk areas of predicted early marriage and the green color indicates the predicted low-risk areas of early marriage. The Amhara, Afar, Gambela, and some parts of the Somali region, were predicted high-risk areas of early marriage. Continuous images produced by interpolating (Kriging interpolation method) early marriage among women (Fig. 6).

### Factors associated with early marriage

After adjusting for different confounding variables, age group, women education level, and region were significantly associated with early marriage in Ethiopia. As the age group increases the odds of early marriage decrease. Women in the age group of 20–34 and 35–49 has a lesser chance of 61% (AOR = 0.39; 95% CI 0.30, 0.51) as compared to the age group of women 15 to 19. The odds of early marriage decreases as the educational level increases. Women who had primary education level has a lesser chance of early marriage by 40% (AOR = 0.60; 95% CI 0.51, 0.71), women who had secondary education level has a lesser chance of early marriage by 81% (AOR = 0.19; 95% CI 0.13, 0.26], women who had higher education level has a lesser chance of early marriage by 89% (AOR = 0.11; 95% CI 0.07, 0.18] as compared to women unable to read and write. The odds of early marriage has a higher chance of 47% (AOR = 1.47; 95% CI 1.16, 1.87) in Amhara and 42% (AOR = 1.47; 95% CI 1.16, 1.87) Gambella Region as compared to women living in Tigray region.The odds of early marriage has lesser chance by 21% (AOR = 0.79; 95% CI 0.63, 0.99) in Oromia, 45% (AOR = 0.55; 95% CI 0.42, 0.70) in Somali, 28% (AOR = 0.72; 95% CI 0.56, 0.92) in SNNP and Harari, 65% (AOR = 0.35; 95% CI 0.25, 0.47) and 31% (AOR = 0.69; 95% CI 0.52, 0.91) as compared to women living in Tigray region (Table 3).

**Table 3:** Multiple logistic regression analysis of factors associated with early marriage among reproductive age in Ethiopia, EDHS 2016

Variables	Marriage year		Crude odds ratio (95% CI)	Adjusted odds ratio (95% CI)
	Under 18 year	Above 18 year		
Place of residence				
Urban	1026	1077	1	1
Rural	6297	3247	2.03 (1.71, 2.42)	0.95 (0.72, 1.18)
Age group				
< 20	966 271	1	1	
20–34	3692	2576	0.41 (0.32, 0.50)	0.39 (0.30, 0.51)*
35–49	2664	4477	0.50 (0.40, 0.63)	0.39 (0.30, 0.51)*
Women level of education				
Unable to read and write	4909	2148	1	1
Primary education	2039	1311	0.68 (0.59, 0.78)	0.60 (0.51, 0.71)*
Secondary education	267	497	0.23 (0.18, 0.30)	0.19 (0.13, 0.26)*
Higher education	106	366	0.12 (0.08, 0.17)	0.11 (0.07, 0.18)*
Husband level of education				
Unable to read and write	3247	1516	1	1
Primary education	3448	1323	0.86 (0.74, 1.03)	1.10 (0.93, 1.30)
Secondary education	466	508	0.42 (0.34, 0.53)	0.89 (0.70, 1.14)
Higher education	241	471	0.23 (0.17, 0.31)	0.82 (0.55, 1.23)
Wealth quartile				
Poor	3043	1559	1	1
Middle	1530	793	0.92 (0.78, 1.08)	0.95 (0.79, 1.14)
Rich	2747	2070	0.63 (0.53, 0.75)	0.98 (0.80, 1.21)
Region				
Tigray	542	304	1	1
Afar	81	27	1.67 (1.25, 2.23	1.25 (0.92, 1.70)
Amhara	2108	778	1.51 (1.19, 1.93)	1.47 (1.16, 1.87)*
Oromia	2751	1681	0.91 (0.72, 1.75)	0.79 (0.63, 0.99)*
Somalia	199	158	0.70 (0.55, 0.89)	0.55 (0.42, 0.70)*
Benishangul Gumuz	81	43	1.04 (0.80, 1.35)	0.94 (0.70, 1.25)
SNNP	343	966	0.77 (0.60, 0.99)	0.72 (0.56, 0.92)*
Gambela	21	12	0.97 (0.75, 1.27)	1.42 (1.02, 1.97)*
Harari	15	13	0.65 (0.50, 0.85)	0.72 (0.55, 0.95)*
Addis Ababa	141	310	0.25 (0.20, 0.31)	0.35 (0.25, 0.47)*
Dire Dawa	34	27	0.69 (0.53, 0.90)	0.69 (0.52, 0.91)*
Media exposure				
No media exposure	5968	3095	1	1
Has media exposure	1353	1229	0.59 (0.49, 0.66)	1.17 (0.97, 1.42)
Occupation status of mothers				
No	3551	2127	1	1
Yes	3771	2196	1.02 (0.95, 1.15)	1.08 (0.96, 1.23)

^*^Significance at 5% level and *CI* confidence interval

## Discussion

This study identified the spatial pattern and its associated factor of early marriage in Ethiopia using hot spot analysis and multiple binary logistic regression model. The hot spot areas of early marriage were located in Amhara, Afar, and Gambella regions of Ethiopia whereas age group, women education level, and region were significant factors associated with early marriage in Ethiopia.

Despite Ethiopia has instituted laws inhibiting marriage under 18 years and its early marriage of girls is associated with a number of poor social and physical outcomes for young women and their offspring [24] child marriage is a norm in the country. Similarly, more than 62% of the study participants in this study were married before they reached the age of 18 years. This finding is higher than the findings from Zambia, 31.4% [25], and Ghana, 29.9% [26]. This might be explained by the disparity in educational, socioeconomic, and cultural differences between the study settings. The problem needs a comprehensive approach, including their families, the community, the government, and religious leaders, to reduce child marriage, teenage childbearing, and its negative consequences.

The spatial autocorrelation result revealed that the spatial distribution of early marriage in Ethiopia was non-random. This means that early marriage is concentrated in some parts of the country. The hot spot analysis of the result revealed that early marriage was high in Amhara, Afar, Gambella regional states of Ethiopia. This finding was consistent in studies conducted in Ghana [27]. The possible justification of a high proportion of early marriage in Amahara, Afar, and Gambela region might be deep-rooted traditions such as, considering marriage as a success for the girl and her family, ensuring the virginity of the girl when she marries, the concern that the girl will become too old for marriage, and creating a bond with the bridegroom's family [13].

This study evidenced that, as the age of mothers increases the likelihood to have an early marriage decreases. This finding is consistent with studies conducted in Ethiopia [10, 28]. The possible justification might be with young women who had married at ages < 20, those married before age 20 were less likely to have known about the disadvantage of early marriage because of teenage [29].

Early marriage is often common among poor and less educated communities. A similar finding is found in this study. Women who attended primary education and above were less likely to marry before 18 years compared to their counterparts. Because educated women have a chance to determine their first age of marriage and are more likely to have a say in decision-making regarding the size of their families and the spacing of their children. In addition, educated women are also likely to be more informed and knowledgeable about contraception and the healthcare needs of their children [30, 31]. But if one’s educational achievement is low, there will be a disconnection of knowledge and information and also fewer youth activities. Moreover, the role of parents in the continuity of early marriage is mainly inseparable from their knowledge linked to their educational achievement. Parents with less understanding of family life may consider early marriage as the best solution to create a better relationship with others [13, 29].

In this study, women were more likely to early marriage in other regions of the country compared to Addis Ababa. It is true that poverty is one of the most powerful drivers of the harmful practices and poor families believed they will be more financially secure once their daughters are married off and out of their responsibility [32]. In addition, poor families want to reduce the number of children to feed, clothe, and educate and families may agree to child marriage because of community pressures and norms [31].

### Strength and limitation of the study

The strong side of this study was having used large country survey data set which was nationally representative. Confounding was controlled by multivariable analysis. The limitation of this study was the cross-sectional nature of the data which may not show true causality. Besides, the dataset does not include important variables like cultural, behavioral, and social norms of the society which had a significant effect on Early marriage.

## Conclusion

Marriage below age 18 is high in Ethiopia. High-risk area of early marriage was located in Amhara, Afar, and Gambella. Governmental and non-governmental organizations should design an effective intervention in these regions to reduce Early marriage. Therefore, providing educational opportunities to young girls was important in addition to inhibiting the marriage of girls under 18 years.

## Data Availability

The datasets generated during the study are publicly available from the Measure DHS Website www.measuredhs.com

## Abbreviations

CI: Confidence interval
EAs: Enumeration areas
EDHS: Ethiopia Demographic and Health Survey
SNNPR: Southern Nation Nationality People of Ethiopia Region
SDG: Sustainable Development Goal
